# Surgical Removal of Impacted Lower Second Molar in Transverse Position: A Case Report

**DOI:** 10.1155/crid/8886597

**Published:** 2025-03-25

**Authors:** Jimmy Antonio Ascanoa Olazo, Miriam Arellanos-Tafur, Felix Rojas-Arquinego

**Affiliations:** ^1^School of Stomatology, Universidad Senor de Sipan, Chiclayo, Peru; ^2^School of Dentistry, Universidad Catolica Santo Toribio de Mogrovejo, Chiclayo, Peru; ^3^School of Stomatology, Universidad Cesar Vallejo, Piura, Peru

**Keywords:** flap extraction, impacted tooth, oral surgery, permanent second molar

## Abstract

**Introduction:** An impacted tooth is one that has not reached the occlusal plane due to a mechanical obstruction, which may include an adjacent tooth, a supernumerary tooth, or an odontoma. Lower third molars are the most frequently impacted teeth, whereas second molars are impacted less often. Currently, diagnostic imaging, such as tomography, is crucial for determining the treatment plan. For the extraction of impacted teeth, a vestibular approach is generally recommended, and for molars, odontectomy prior to tooth luxation and avulsion is advised.

**Objective:** This study was aimed at describing the surgical technique for the flap extraction of an impacted lower second molar in a transverse position with the crown oriented towards the lingual side.

**Case Presentation:** A 17-year-old male patient presented with pain in the lower left molar region. Clinical examination revealed the absence of Teeth 37 and 38. Tomographic imaging showed Tooth 37 in a transverse position with the crown oriented lingually and Tooth 38 in a vertical position. Extraction of Tooth 37 was performed using a vestibular approach and odontectomy due to space constraints.

**Conclusions:** Retention of permanent second molars is rarely reported in the literature. It is advisable to apply all possible methods to position these teeth in the occlusal plane to ensure proper masticatory function. However, there are cases where extraction is necessary due to space limitations, and alternative solutions for replacing the lost tooth should be explored.

## 1. Introduction

Dental eruption is defined as “the axial or occlusal movement of a tooth from its developmental position within the jaw to its functional position in the occlusal plane” [1]. This process continues over time as compensation for occlusal wear and jaw growth, with changes occurring during facial growth and development. Insufficient eruption can result in positional anomalies and malocclusions [2].

Disruptions in the eruption process can lead to consequences such as impaction, ectopic eruption, primary and secondary retention, supernumerary teeth, odontomas, and failure of the eruption mechanism. These disruptions can occur at any stage of eruption due to an obstacle in the eruption path [3, 4]. Permanent second molars (PSM) erupt around the age of 12 [5], and studies confirm that their impaction is more common in males than in females [6].

Disorders in the eruption of maxillary and mandibular molars are associated with mechanical obstructions, including hyperdontia, tumors, cysts, mesial eruption, subsequent impaction in the distal region of the adjacent tooth, dentoalveolar discrepancies, increased space between the developing second molar and the first molars, and abnormal eruption trajectories [7, 8].

Impaction is an unusual clinical finding due to its sporadic occurrence [9]. The prevalence of impaction in mandibular molars was reported by Fu et al. [10] and Cassetta et al. [11] as 0.65% and 1.36%, respectively. Occasionally, mesial angulation of the lower PSM, due to uneven root growth, may cause impaction, complicating normal and functional eruption [12].

Diagnosis of second molar impaction is typically performed using periapical and panoramic radiographs. This condition may not be detected until well after the expected eruption time [13]. Extraction of the impacted lower second molar is recommended if repositioning is deemed unfeasible due to its unfavorable position in the mandibular arch [14]. Consequently, managing impacted molars can be challenging, as spontaneous eruption failure may necessitate surgical interventions.

Currently, extraction of impacted molars remains one of the most frequently performed procedures in oral and maxillofacial surgery [15]. Therefore, the aim of this study was to describe a clinical case involving the surgical removal of a horizontally impacted lower second molar with the occlusal face oriented lingually.

## 2. Case Report

**Figure 1:**
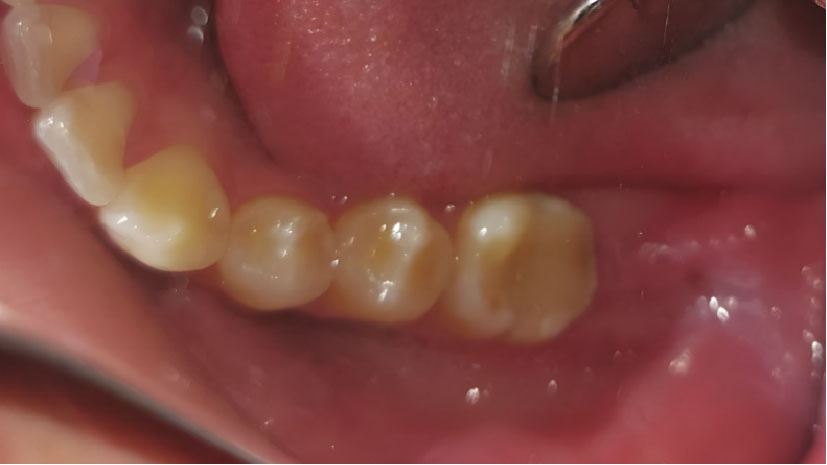
Clinical examination showed the absence of Teeth 37 and 38.

A 17-year-old male patient presented with pain in the lower left posterior region of the mandible. The patient had no significant systemic history and reported no previous extractions in the mentioned area. Clinical examination revealed the absence of Teeth 37 and 38. Palpation of the alveolar ridge showed no swelling or bony prominences (Figure 1).

**Figure 2:**
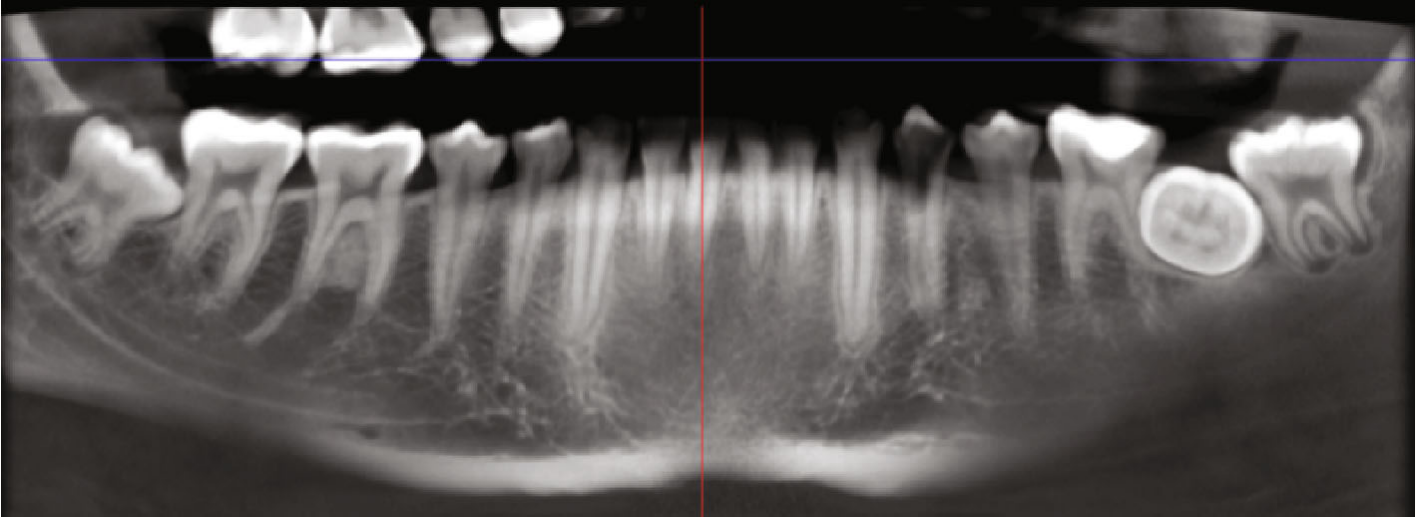
Panoramic view: Tooth 37 impacted in a transverse position.

**Figure 3:**
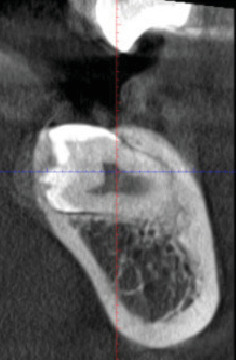
Tomographic coronal section: Tooth 37 in transverse position with the crown oriented lingually.

**Figure 4:**
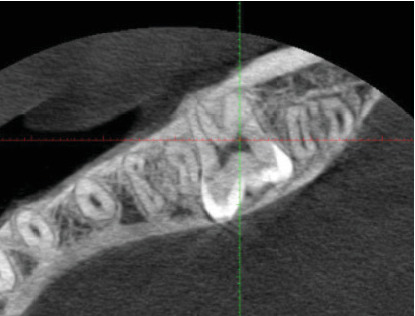
Tomographic axial section: Tooth 37 with the crown oriented lingually.

**Figure 5:**
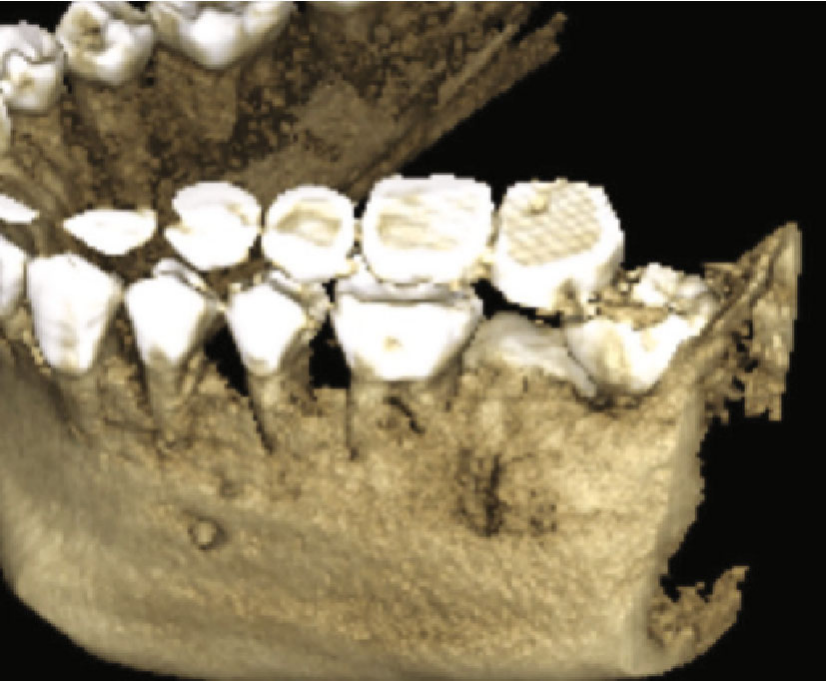
3D reconstruction tomography view of Tooth 37 in a transverse position with the crown oriented lingually.

Radiographic examination revealed that Tooth 37 was impacted transversely between Teeth 36 and 38, with Tooth 38 in a vertical position close to the occlusal plane, Stage Nolla 8 (Figure 2). Tomographic imaging indicated that the crown of Tooth 37 was oriented lingually, with the roots separated; the mesial root was curved and thin, while the distal root was straight and thick (Figures 3, 4, and 5).

**Figure 6:**
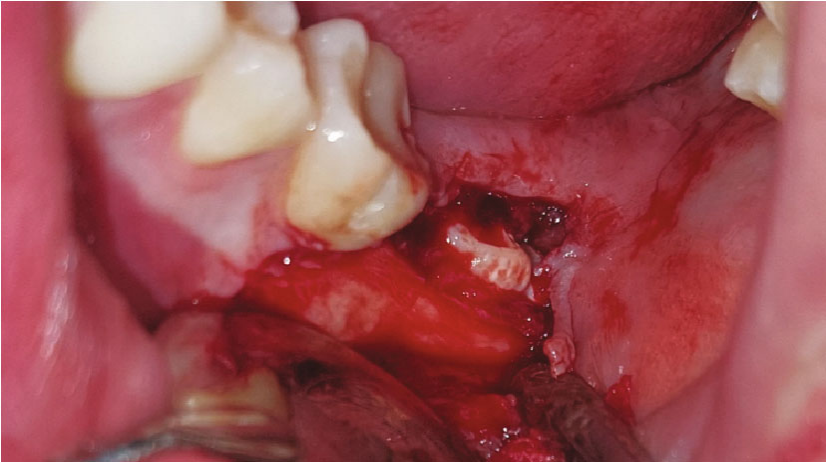
Flap elevation and view of Tooth 38. Tooth 37 is not yet visible.

**Figure 7:**
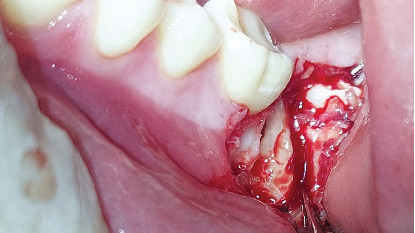
Sectioned roots of Tooth 37.

The diagnosis was an impacted Tooth 37 in a transverse position with the crown oriented lingually and Tooth 38 retained in a vertical position. A consultation with the orthodontics department was made to define the treatment plan. Due to the position of the second molar and the lack of space for repositioning, flap extraction of Tooth 37 was planned, with subsequent eruption of Tooth 38 and fixed orthodontic treatment. The ultimate goal was to restore masticatory function by positioning the third molar in place of the second molar. The patient was informed regarding the surgical procedure, the risks associated with it, and the alternative treatments. He signed the informed consent through which he agreed to enter the study and to the use of images of the surgical procedure in scientific articles. The surgical intervention for flap extraction of Tooth 37 was scheduled. The procedure involved block anesthesia for the inferior alveolar nerve, lingual nerve, and buccal nerve. An L-shaped or monoangular incision was made at the level of the second molar. The mucoperiosteal flap was elevated to expose the bone plate (Figure 6). An osteotomy was performed in the occlusal bone to visualize the crown of the second molar. The separated roots were sectioned for removal due to the limited space between the first molar and the third molar and the width of the second molar crown (Figure 7).

**Figure 8:**
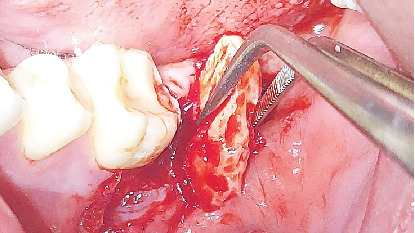
Removal of the distal root.

**Figure 9:**
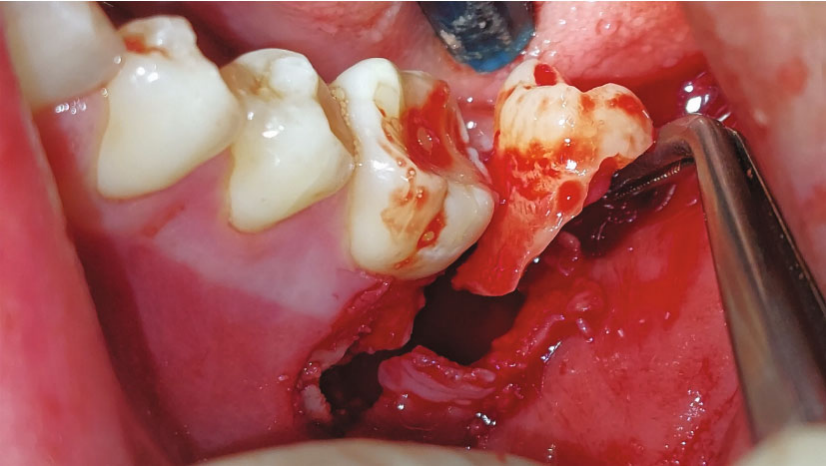
Removal of the mesial root.

The distal segment (crown and root) was extracted first due to better visibility during the process, followed by removal of the mesial segment (crown and root) (Figures 8 and 9). Both segments were luxated with a flag elevator.

**Figure 10:**
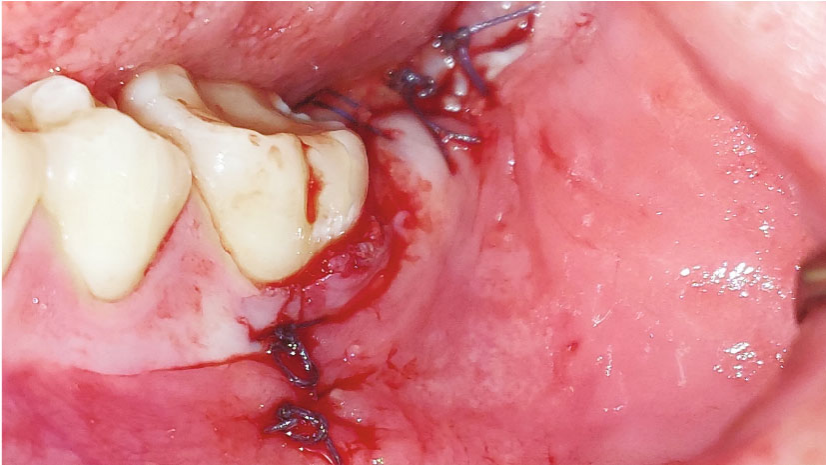
Discontinuous suturing with 3/0 polyglycolic acid, five simple sutures.

**Figure 11:**
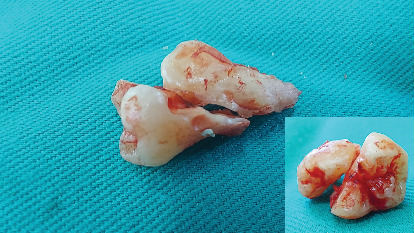
Extracted molar.

An alveolar curette was used to clean the bony crypt corresponding to the crown and remove the pericoronal sac, followed by irrigation with 0.9% sodium chloride. The procedure was completed with discontinuous suturing using 3/0 polyglycolic acid, with five simple sutures (Figure 10). The extracted molar was observed in two parts (Figure 11). Amoxicillin 500 mg every 8 h for 5 days and sodium diclofenac 50 mg and dexamethasone 4 mg both every 8 h for 3 days were prescribed.

Regarding follow-up, the patient was evaluated 6 months after the surgical intervention with favorable bone healing according to an x-ray. The report confirms “alveolar walls preserved, no evidence of spicules or bony defects; defined borders, no radiographic signs of osteitis” (see Figure S1).

## 3. Discussion

Dental eruption is a biological process in postnatal development, and it is related to the formation of the root as a critical structure anchored to the surrounding alveolar bone through the periodontal ligament [16, 17]. Alterations in the eruption of a PSM are relatively rare [18]. Proper dental development, coordinated facial growth, and occlusion are crucial for the first and second permanent molars [19]. In this case, impaction of a lower PSM resulted in reduced masticatory forces and potential extrusion of the antagonist's tooth, posing a risk of occlusal dysfunction.

Impaction of second molars is uncommon; the reported case is infrequent in clinical practice, necessitating a multidisciplinary approach for diagnosis and treatment planning. A study on second molars found the prevalence of eruption disturbances, agenesis, ectopic eruption, and impacted teeth to be 2.3%, 0.8%, 1.5%, and 0.2%, respectively [11].

Stewart et al. [20] report a low frequency of agenesis of primary and permanent teeth, confirming the unusual incidence. Additionally, Gay and Berini [21] mention that the causes of retention and impaction in molars are due to space constraints related to human evolution.

Incomplete or partial eruption and adjacent positioning of a tooth to another tooth, bone, or soft tissue significantly impede the eruption process, according to its anatomical location [22]. In unilateral impaction of the lower second molar, the issue is often associated with altered spatial relationships in the posterior mandible.

Tooth 37 in the transverse position presented diagnostic and therapeutic challenges. Clinical examination alone could not determine the position of the tooth or any relationship with adjacent structures. Accurate diagnosis was made through imaging, including panoramic radiographs and tomographic images [23]. The case described aligns with the findings of Arunachalam et al. [24], who report that the most frequent positions for lower molar impactions are vertical, horizontal, and mesioangular.

Surgical removal of impacted molars is indicated when conservative treatment fails. For Tooth 37, flap extraction with an L-shaped incision, osteotomy, and tooth sectioning allowed successful extraction despite the complex positioning of the tooth.

## 4. Conclusion

The extraction of impacted lower second molars, particularly in transverse positions, presents a complex challenge due to the potential for significant anatomic variation and the need for careful surgical planning. Diagnostic imaging plays a critical role in determining the appropriate surgical approach. The case highlights the importance of individualized treatment plans and the role of surgery in managing difficult tooth extractions. This approach ensures restoration of function and prevents potential complications associated with impacted teeth.

## Data Availability

The data that support the findings of this study are available on request from the corresponding author. The data are not publicly available due to privacy or ethical restrictions.
