# Supplementary material for: Surgical Removal of Impacted Lower Second Molar in Transverse Position: A Case Report
Source: Case Rep Dent. 2025 Mar 25;2025:8886597. doi: 10.1155/crid/8886597 (PMC11961287; doi:10.1155/crid/8886597)
Supplement: Supporting Information — Additional supporting information can be found online in the Supporting Information section. Figure S1: Bone healing after 6 months. [file 8886597.f1.docx]

**Supplementary material**

After the removal of the stitches, the patient was told that he should continue his treatment with an orthodontic specialist in order to place the tooth 38 in the correct position after its eruption. However, the patient could not continue with this treatment because he moved to another city for study purposes. We were able to contact the patient six months after the intervention, and he reported that tooth 38 erupted and due to discomfort it was extracted. A control x-ray was indicated to verify the bone healing, the report mentions that “*alveolar walls preserved, no evidence of spicules or bony defects; defined borders, no radiographic signs of osteitis*”.(Fig.1)


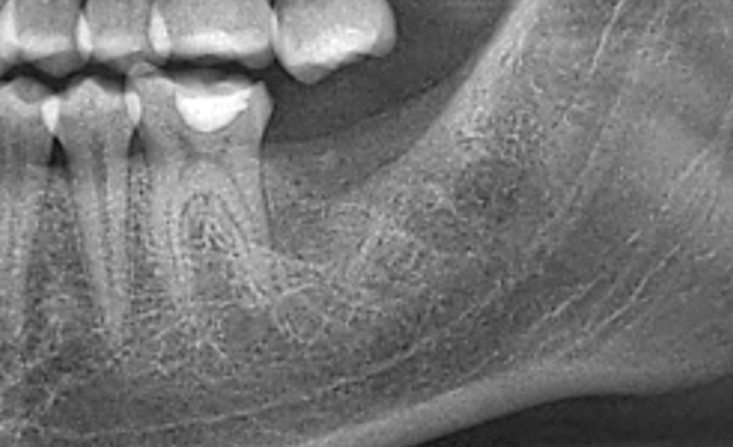


Figure 1. Bone healing after six months
